# An unusual presentation of diabetic ketoacidosis associated with ascariasis and fungal esophagitis: A case report

**DOI:** 10.1097/MD.0000000000032015

**Published:** 2022-11-25

**Authors:** Rabia Khalid Alduraibi, Elzaki Mohamed Elzaki, Ammar Alammari

**Affiliations:** a Department of Endocrine and Diabetes, King Fahad Specialist Hospital, Buraydah, Saudi Arabia; b Department of Gastroenterology, King Fahad Specialist Hospital, Buraydah, Saudi Arabia.

**Keywords:** ascariasis, diabetic ketoacidosis, fungal esophagitis, type 1 diabetes

## Abstract

Gastrointestinal symptoms are most commonly observed in patients with diabetic ketoacidosis (DKA), which usually resolves completely with therapy. However, gastrointestinal symptoms may persist after DKA is resolved.

**Patient concerns:**

A 22-year-old female was admitted to hospital for epigastric pain and persistent vomiting. The results of laboratory examination showed fungal esophagitis complicated by DKA.

**Diagnosis:**

The patient was diagnosed with DKA associated with ascariasis and fungal esophagitis

**Outcome:**

The patient was discharged after treatment

**Lessons:**

In this case, despite the correction of metabolic acidosis, persistent nausea, vomiting and dysphagia can be a sign of esophagitis in patients with type 1 diabetes. Therefore, physicians should be aware of fungal infections associated with type 1 diabetes.

## 1. Introduction

Diabetic ketoacidosis (DKA) is a life-threatening endocrine emergency that requiring admission to the intensive care unit. The development of DKA has been associated with several precipitating factors such as infection, ischemia, medications, and other medical-surgical conditions. The 2 extremely rare infections that cause DKA are ascariasis and candidiasis.

Here, we report the case of a patient with type 1 diabetes who presented with epigastric pain and persistent vomiting and was finally diagnosed with ascariasis and fungal esophagitis complicated by DKA.

To the best of our knowledge, this is the first case report of multifactorial DKA in Saudi Arabia.

## 2. Case presentation

**Figure 1.**
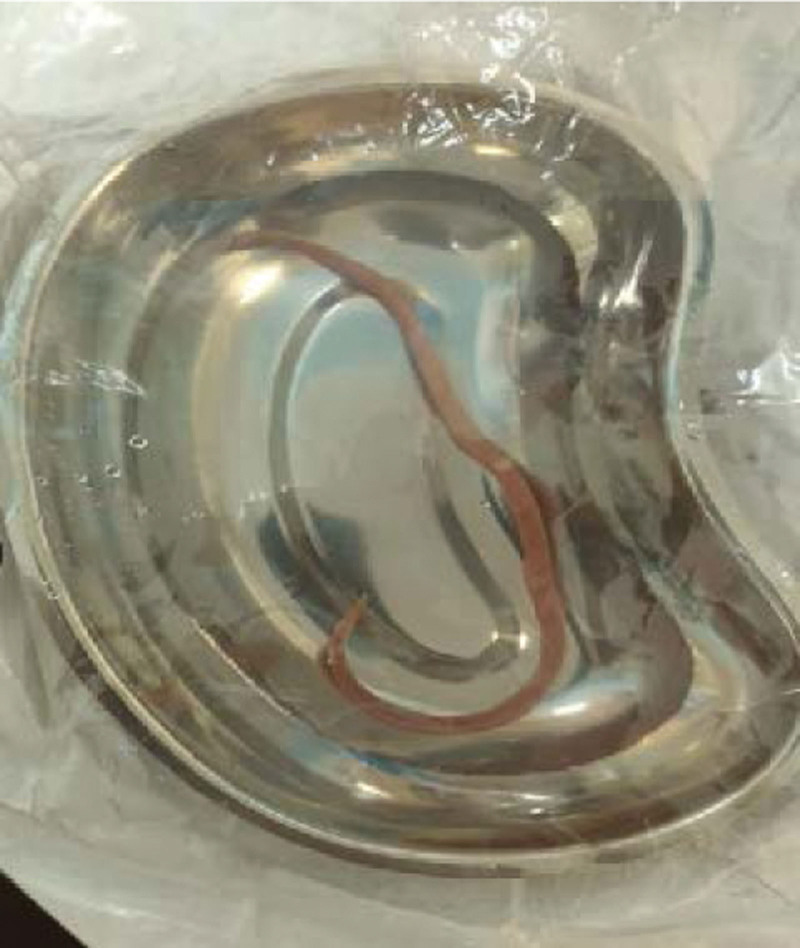
Round worm vomited by the patient, approximately 18 cm in length and 5 mm in diameter with curved tail typical of a male worm.

**Figure 2.**
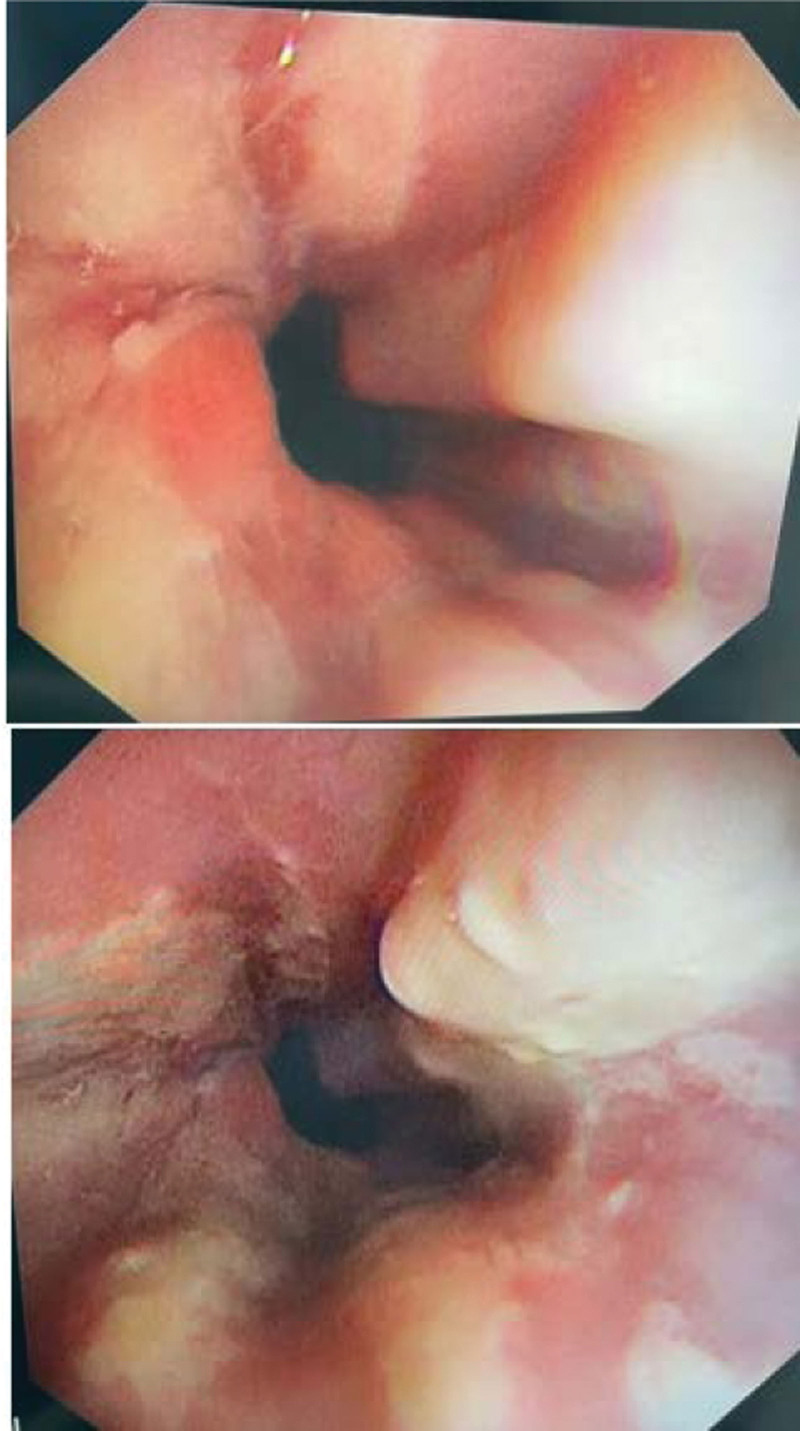
Endoscopic showing severe esophagitis with extensive white patches in the middle and lower esophagus.

A 22-year-old Pakistani female residing in Saudi Arabia has been known to have type 1 diabetes mellitus since the age of 19 years. The patient presented to our hospital with abdominal pain associated with nausea and vomiting. Initial laboratory test results revealed diabetic metabolic acidosis. Despite the prompt correction of ketoacidosis, her nausea and vomiting persisted until 1 day, the patient vomited an 18-cm round worm, as shown in Figure 1. She was treated with antiparasitic and antiemetic drugs, but still complained of persistent vomiting. She denied any of the following symptoms: oral thrush, dysphagia, odynophagia, abdominal distension, diarrhea, constipation, or unintentional weight loss. Physical examination revealed mild tenderness in the epigastric region. Enhanced abdominal computed tomography was performed to rule out intestinal obstruction, which was unremarkable. Furthermore, she underwent an upper gastrointestinal endoscopic examination, which revealed severe esophagitis with significant white patches in the middle and lower esophagus, as shown in Figure 2. Mild-to-moderate gastritis without antral nodularity was observed. The duodenum is normal.

**Figure 3.**
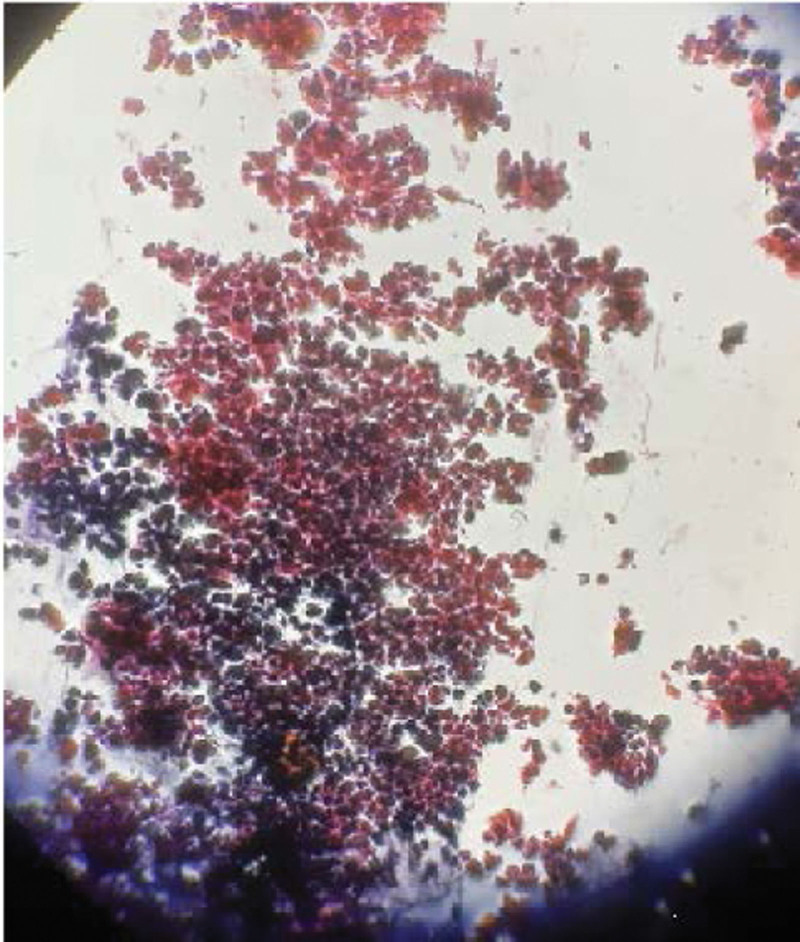
Histological examination showing pseudohypal fungal forms (black arrow).

Histopathological examination of endoscopic biopsies confirmed that the patient had chronic esophagitis and gastritis without evidence of celiac disease or Helicobacter pylori infection. Several fungal spores were found in the esophageal biopsies, which were eventually identified as Candida, as shown in Figure 3. Fluconazole was administered to treat the patient. The patient’s condition improved significantly thereafter.

This is one of the few case reports of ascariasis combined with Candida esophagitis complicated with DKA. Therefore, in regions where ascariasis is geographically endemic, ascariasis and Candida esophagitis are possible etiologies for DKA.

## 3. Discussion

Helminth parasites and infective esophagitis are rare diseases that mostly affect adults with diabetes in tropical and developing countries where sanitation facilities are unsatisfactory. Additionally, they are considered rare precipitating factors of DKA.

To the best of our knowledge, this is an uncommon presentation of ascariasis combined with fungal esophagitis complicated with DKA, underscoring the importance of endoscopy and biopsy.

Fungal esophagitis was reported in 14 (27.4%) adult patients with uncontrolled diabetes in Pakistan.^[1]^ There is 1 case report of Candida esophagitis in a pediatric patient with type 1 diabetes in Saudi Arabia.^[2]^

In this report, we described a patient with type 1 DM who presented with DKA and ascariasis. Her symptoms persisted despite treatment with proton pump inhibitors and antiparasitic drugs. Endoscopic examination revealed fungal esophagitis.

The presentation of esophageal candidiasis in immunocompetent patients includes odynophagia, dysphagia, heartburn, and retrosternal discomfort. More severe cases can cause systemic candidiasis, esophageal dysmotility, stricture, fistulae and perforation.^[2–4]^

Typical features of endoscopic findings are the presence of mucosa in the form of white plaques, which are difficult to wash, and brushing that causes the mucosa to bleed but does not wash it away.^[5]^ Hyphae or spores present in an esophageal biopsy specimen are diagnostic features of Candida esophagitis.

Esophageal candidiasis is found only in patients with uncontrolled diabetes who are noncompliant with either their diet or medication, as in this case. Two- to 6-week courses of antifungal drugs therapy, such as fluconazole, are sufficient to eliminate the fungal infection.^[2]^

## 4. Conclusion

In conclusion, persistent nausea, vomiting, and dysphagia despite correction of metabolic acidosis could be a sign of esophagitis in patients with type 1 diabetes. Therefore, physicians should be aware of fungal infections associated with type 1 diabetes.

## Acknowledgments

We are grateful to the patient and her family who kindly consented to participate in the study.

## Author contributions

RD collected the data, drafted the initial manuscript, and reviewed the manuscript. EE and AA coordinated and supervised data collection and reviewed the manuscript. All the authors have read and approved the manuscript.

**Conceptualization:** Rabia Khalid Alduraibi.

**Data curation:** Rabia Khalid Alduraibi, Elzaki Mohamed Elzaki, Ammar Alammari.

**Investigation:** Rabia Khalid Alduraibi.

**Methodology:** Rabia Khalid Alduraibi.

**Project administration:** Rabia Khalid Alduraibi.

**Supervision:** Rabia Khalid Alduraibi, Elzaki Mohamed Elzaki, Ammar Alammari.

**Writing – original draft:** Rabia Khalid Alduraibi, Elzaki Mohamed Elzaki, Ammar Alammari.

**Writing – review & editing:** Rabia Khalid Alduraibi, Elzaki Mohamed Elzaki, Ammar Alammari.
